# Rac1 Signaling: From Intestinal Homeostasis to Colorectal Cancer Metastasis

**DOI:** 10.3390/cancers12030665

**Published:** 2020-03-12

**Authors:** Larissa Kotelevets, Eric Chastre

**Affiliations:** 1 Institut National de la Santé et de la Recherche Médicale, UMR S 938, Centre de Recherche Saint-Antoine, 75012 Paris, France; 2 Sorbonne Université, Hôpital Saint-Antoine, Site Bâtiment Kourilsky, 75012 Paris, France

**Keywords:** Rac1, small GTPase, colorectal cancer, intestine, E-cadherin, Wnt signaling, epithelial mesenchymal transition, invasiveness, metastasis, alternative splicing

## Abstract

The small GTPase Rac1 has been implicated in a variety of dynamic cell biological processes, including cell proliferation, cell survival, cell-cell contacts, epithelial mesenchymal transition (EMT), cell motility, and invasiveness. These processes are orchestrated through the fine tuning of Rac1 activity by upstream cell surface receptors and effectors that regulate the cycling Rac1-GDP (off state)/Rac1-GTP (on state), but also through the tuning of Rac1 accumulation, activity, and subcellular localization by post translational modifications or recruitment into molecular scaffolds. Another level of regulation involves Rac1 transcripts stability and splicing. Downstream, Rac1 initiates a series of signaling networks, including regulatory complex of actin cytoskeleton remodeling, activation of protein kinases (PAKs, MAPKs) and transcription factors (NFkB, Wnt/β-catenin/TCF, STAT3, Snail), production of reactive oxygen species (NADPH oxidase holoenzymes, mitochondrial ROS). Thus, this GTPase, its regulators, and effector systems might be involved at different steps of the neoplastic progression from dysplasia to the metastatic cascade. After briefly placing Rac1 and its effector systems in the more general context of intestinal homeostasis and in wound healing after intestinal injury, the present review mainly focuses on the several levels of Rac1 signaling pathway dysregulation in colorectal carcinogenesis, their biological significance, and their clinical impact.

## 1. Introduction

Colorectal cancer (CRC) is a major cause of cancer morbidity and mortality in western countries. Nearly 140,000 and 450,000 individuals are diagnosed annually in United-States and in Europe, where this cancer is responsible of approximately 53,000 and 215,000 related deaths, respectively [1,2]. Colorectal cancers arise through the stepwise accumulation of genetic alterations, including the activation/overexpression of a series of (proto)-oncogenes, and the inactivation of tumor suppressor genes [3,4] and follow three molecular pathways to genome instability characterized by (i) chromosomal instability (CIN), (ii) high microsatellite instability (MSI-H), or (iii) CpG island methylator phenotype (CIMP). Mutations in *APC*, *TP53*, *KRAS*, *PIK3CA*, *FBXW7*, *SMAD4*, *TCF7L2*, and *NRAS* genes are the most frequently identified in CRC (cancer genome atlas network 2012). Activating mutations of *KRAS*, *NRAS*, and *HRAS* genes occurs in 33%, 3.7%, and 0.9% of CRC, respectively (URL http://cancer.sanger.ac.uk/, COSMIC v90, released 5 September 2019). Ras proteins belong to a superfamily of small GTPases composed of Ras, Rho, Ran, Rab, and Arf. These GTPases act as binary switches from active GTP (Guanosine triphosphate)-bound form, that interacts with effector molecules to initiate signaling, to the GDP (Guanosine diphosphate)-bound inactive form.

**Figure 1:**
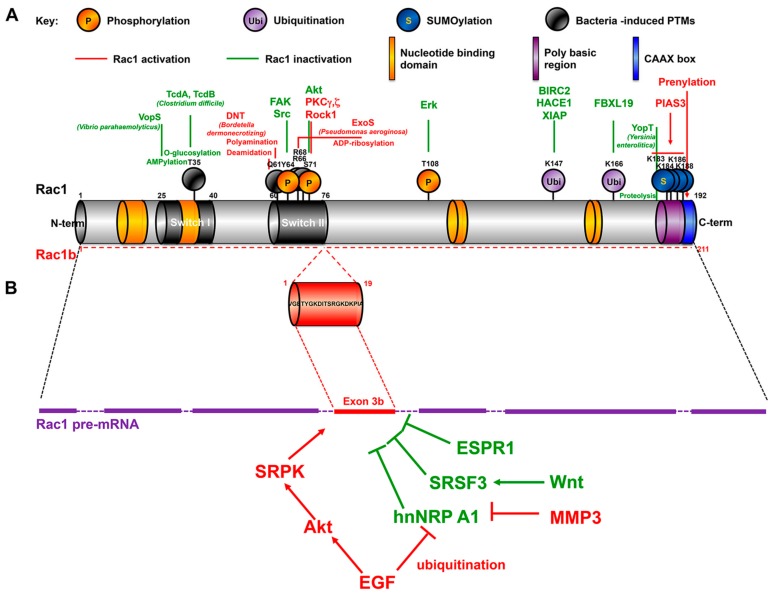
Primary structure of Rac1 and Rac1b splice variant. (**A**) Posttranslational modifications (PTMs) of Rac1. In green are represented PTMs that inactivates Rac1, in Red those that stimulates the GTPase signaling. (**B**) Regulation of Rac1 splicing. For details see the text.

**Figure 2:**
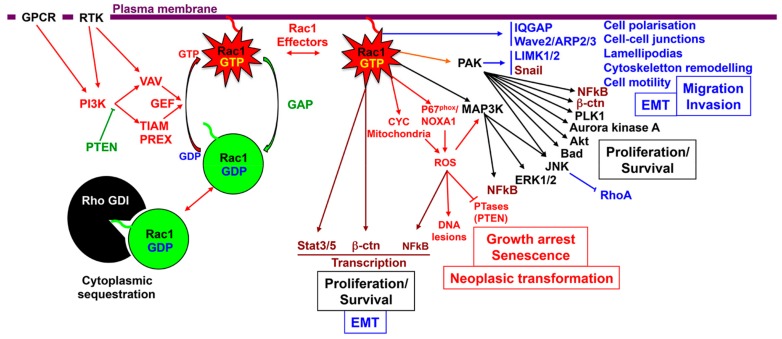
Schematic representation of Rac1 signaling pathways and their biological significance. Rac1 upstream regulators: the Rac1 activating and inhibitory pathways are represented in red and in green respectively. Rac1 interacting partners and downstream effectors: transcription factors are represented in brown, effector molecules in black, pathways involved in cytoskeleton remodeling and cell migration in blue, and ROS pathway in Red.

The Rho family of small GTPases are involved in the regulation of actin cytoskeleton remodeling, cell polarity, cell adhesion and migration, but also other processes including stem cell maintenance, cell proliferation and differentiation. Among the twenty members of this family, the best characterized GTPases are RhoA, Rac1, and Cdc42. Rac GTPases encompass 4 members: Rac1 which is ubiquitous, Rac2 mainly expressed in hematopoietic cells, Rac3 expressed in brain and testis, and RhoG present in fibroblasts, leukocytes, neuronal, and endothelial cells [5]. The GTP-bound state of Rac1 is accompanied by a conformational change in two regions, termed switch I and II (encompassing amino acids 25–40 and 60–76, respectively), which allows the selective interaction with diverse effectors that mediate downstream signaling cascade (Figure 1 and Figure 2). The activity of Rac1 is positively regulated by Guanine nucleotide Exchanges Factors (GEFs) favoring the GDP/GTP exchange, GTPase Activating proteins (GAPs) favoring the switch on/off (GTP/GDP), and Guanosine nucleotide Dissociation Inhibitor (GDI) which binds to the GDP-bound forms, preventing the GDP/GTP exchange (off-state) but also sequestering the small GTPase in the cytoplasm. More than 80 GEFs and 70 GAPs for the Rho GTPase family have been identified, highlighting the fine tuning of the level and the activity of the GTPases, and of the assembly and subcellular targeting of scaffolds involving these GTPases and their effector systems, to allow the selective activation of signaling cascades and to trigger appropriate cellular response [6]. Accordingly, many Rac1-GEFs are multi-domain proteins allowing the organization of signalosomes and driving their subcellular localization. For instance, the pleckstrin homology (PH) domain, that binds phosphatidylinositol 3,4,5 trisphosphate, in Tiam1, P-Rex1, and Vav1 allows the plasma membrane recruitment of these Rac1-selective GEFs following the activation of receptor tyrosine kinase and the downstream PI3 kinase activity. The selectivity of the downstream effectors driven by these GEFs is exemplified by the different interactomes involving Tiam1 and P-Rex1 that trigger two opposing Rac1 migratory responses. Tiam-1 proved to stabilize junctional complexes, whereas P-Rex1 stimulates cell motility [7,8].

The Rho family of GTPases is also regulated by posttranslational modifications, including prenylation of the C-terminal CAAX motif favoring membrane interaction, but also phosphorylation, SUMOylation, ubiquitination (Table 1, Figure 1 and see below).

Rac1 initiates several signaling pathways, -including PAKs, NOX1 complex/reactive oxygen species (ROS) production, NFkB, members of MAPKs, Wnt/β-catenin/TCF, STAT3-, regulating membrane ruffling, cytoskeletal remodeling, cell adhesion, cell–cell contacts, cell polarity, cell motility, and transcriptional activity, leading to cell proliferation, epithelial mesenchymal transition (EMT), and invasiveness (Figure 2). Thus, this GTPase might be involved at different steps of the neoplastic progression from dysplasia to the metastatic cascade.

The hallmark of carcinogenesis relies on cancer cell invasiveness and dissemination. Rac1 activation and inactivation control plasticity of tumor cell movement [9]. Cancer cell invasiveness might involve individual (mesenchymal, amoeboid) or multi-cellular mode of migration. These modes of cell movement are interconvertible. Mesenchymal migration is driven by Rac1 via the WAVE regulatory complex that activates Arp2/3-dependent actin polymerization to generate membrane protrusions. Blocking Rac1 signaling suppresses mesenchymal movement and enhances amoeboid movement [9]. Amoeboid migration relies more on RhoA-ROCK signaling to promote actomyosin contractility and mechanical forces to move forward. This mode of moving takes advantage of the interstitial spaces, does not require extracellular matrix degradation or strong adhesion, and is independent from proteases and integrins. Collective migration is more representative of differentiated epithelial tumors, although some switch between these different modes of moving may timely operate [10].

## 2. Rac1 in Intestinal Physiology

### 2.1. Role of Rac1 in Intestinal Homeostasis 

The implication of Rac1 in cell proliferation, cytoskeletal dynamic, junctional complexes activity, and cell motility make this small GTPase a critical effector in the maintenance of intestinal barrier integrity under physiological conditions and during tissue repair.

**Figure 3:**
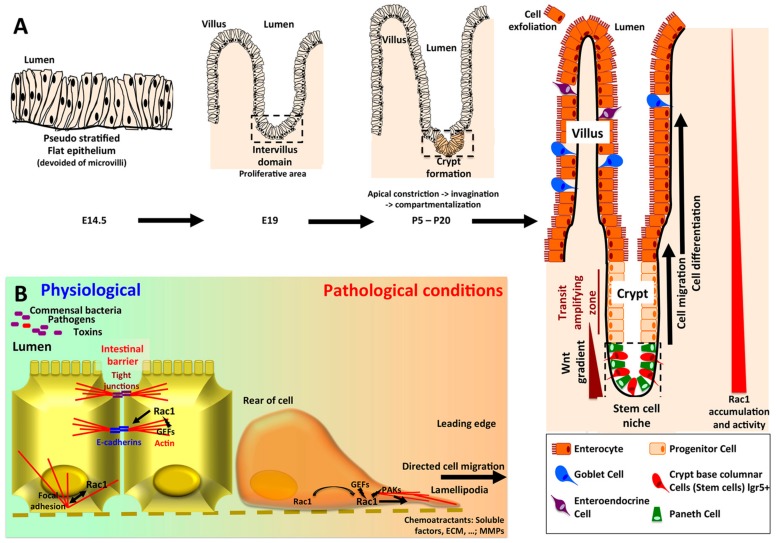
(**A**) Ontogeny of the crypt-villus unit. The intestinal epithelium originates from endoderm, whereas the underlying mesenchymal and muscular layers derive from mesoderm. Intestinal ontogenesis follows an anteroposterior axis. In mouse, at embryonic day 14.5, the intestinal epithelium is pseudostratified and subjected to an active proliferation. By embryonic day 18, the villi have emerged in the duodenum and the proliferative area is restricted to the intervillus domain [64]. The crypts began to invaginate by day 5 post partum. First, myosin II-mediated apical constriction allows invagination of the crypt progenitor cells. Subsequently, a hinge region forms between crypts and villi. This process requires Rac1, which locally acts to suppress hemidesmosomal integrins, allowing cell shape changes and the formation of the hinge cells, leading to the compartmentalization of the crypt and villi [50]. In adult intestine, the stem cells -colocalized with Paneth cells in the bottom of the crypt- give rise to progenitor cells which will mainly differentiate into enterocytes, but also in goblet and enteroendocrine cells, and migrate along the villi to exfoliate at the top, whereas downward cell migration and differentiation allows to renew Paneth cells pool [65]. Wnt3 produced by Paneth cells, as well as EGF and Notch stimuli are required for the maintenance of stem cells. In the crypt villus unit, Rac1 expression and activity follow an increasing gradient from the top of the villi to the bottom of the crypts. As regards the colonic epithelium, the main characteristics rely on the absence of villi (flat mucosa), the absence of Paneth cell (substituted by Reg4+ deep crypt secretory cells), and the main type of differentiation being goblet cells [65]. (**B**) Rac1 in intestinal homeostasis and intestinal barrier repairs. By initiating the formation of E-cadherin complexes, Rac1 plays a critical role in the maintenance of tissue integrity. Upon wound formation, Rac1 localized to the leading edge of the cells, trigger lamellipodia formation through actin cystoskeleton remodeling, and directed cell migration.

In human, the ontogeny of intestine occurs early during embryogenesis. In contrast, in mouse small intestine, the invagination of the intervillus epithelium as a specialized crypts compartment that contains stem cells, Paneth cells and progenitor cells occurs after birth (Figure 3A). A recent study uncovered the involvement of Rac1 signaling in this process. Conditional knockout of Rac1 in the intestine revealed that this GTPase is not required for initial crypt invagination, but later on, Rac1 depletion results in crypt expansion at the expense of villi, mimicking colon architecture [50]. The defect is not secondary to changes in cell proliferation or migration, but seems to be related to changes in cell shape at the crypt/villus boundaries due to increased hemidesmosomal α6/β4 integrin accumulation [50,51].

In adult rat intestine, Rac1 protein levels as well as its GEF Tiam1 gradually increases from the villus tip to crypt compartment (Figure 3A) [52,53]. In this connection, a mouse Rac-FRET model further demonstrated that Rac1 activity is higher in cells at the base of the crypts than in distal cells [54]. Interestingly, the Leucine-rich repeat-containing G protein–coupled Receptor 5 (LGR5), a receptor for R-spondins strengthens cell–cell adhesion of stem cells through IQGAP/Rac1 interaction. LGR5 is a marker of adult stem cells, notably in the intestinal crypts and colon cancer cells that potentiates Wnt signaling via its interaction with the Wnt receptor frizzled. LGR5 favors Rac1-GTP in its competition with β-catenin to interact with IQGAP1, leading to enhanced linkage of E-cadherin junctional complexes to the cytoskeleton. This strong cell-cell adhesion has been proposed to retain stem cells within their niche at the crypt base [55]. Rac1 also enhances the nuclear formation of the β-catenin and LEF-1 complexes, providing the basis for the role of Rac1 in augmenting transactivation of Wnt target genes [56]. The Wnt/β-catenin pathway is a fundamental cornerstone for intestinal epithelial progenitor cell proliferation [57]. The dysregulation of this pathway occurs at high incidence and in the early steps of the neoplastic progression of intestinal epithelium. In mammalian as well as in drosophila, Rac1 proved to be necessary and sufficient to drive intestinal stem cells proliferation and regeneration in a ROS-dependent manner, suggesting the evolutionarily conserved role of Rac1 in intestinal homeostasis [58,59]. In these flies, the Rac1-JNK pathway controls the engulfment of dying intestinal stem cells by neighboring enterocytes [60].

Rac1 proved also to be involved in post-mitotic cells to control their terminal differentiation program. The expression of a constitutively active Rac1 mutant (Rac1Leu61) in mouse chimera induces precocious differentiation of members of the Paneth cells and enterocytic lineages, without affecting cell proliferation. It is worth noting that Paneth cells constitute the niche for Lgr5 stem cells in intestinal crypts [61,62]. On the other hand, the expression of a dominant negative form of Rac1 (Rac1Asn17) results in an inhibition of epithelial cell differentiation and a decrease in cell migration along the crypt–villus axis. Interestingly, none of these Rac1 mutations affect cell proliferation [63].

### 2.2. Intestinal Barrier Integrity/Mucosal Repair/Colitis

Intestinal epithelium does not only constitute a physical barrier against pathogen invasion but it also regulates nutrient uptake and innate immune function by avoiding the activation of mucosal immune responses. The barrier function in intestinal epithelia is carried out by specialized intercellular junctions, including tight junctions, zonula adherens, and desmosomes (Figure 3B). Tight junctions provide the paracellular permeability seal, through transmembrane proteins such as occludins and claudins, which are anchored to the actin cytoskeleton via scaffolding complexes of PDZ-containing proteins. Adherens junctions are composed by E-cadherin transmembrane proteins, anchored to the cytoskeleton through α-catenin, β-catenin, and p120^ctn^. Desmosomes containing desmocollin and desmoglein as transmembrane proteins, are essential to provide tissue integrity and to strength cell–cell junctions. These junctional complexes also participate in outside-in signaling, intestinal epithelial cell polarization, and differentiation [66].

The role of Rho/Rac1/CDC42 in intestinal barrier integrity is illustrated by the selective inhibition of these small GTPases by bacterial toxins leading to actin depolymerization, loss of cell-cell contacts, recruitment of immune cells and diarrhea (Figure 1A, Table 1) [67].

The activity of Rac1 is spatially and temporally orchestrated by scaffolding molecules, GEF and GAP regulatory proteins and effector proteins [68,69,70,71]. This concept is clearly demonstrated by the requirement of Rac1 for the initiation of the formation of E-cadherin-mediated cell-cell adherens junctions, which is linked to reduced cell motility and invasion.

Besides its critical role in the maintenance of cell-cell contacts and apical-basolateral cell polarization, a switch in Rac1 biological function from cell adherence to cell migration occurs following intestinal mucosal injury (Figure 3B). Rac1 triggers the formation of lamellipodia protrusions and epithelial cell migration at the leading-edge of a wound to promote wound closure. During this process, engagement of integrins with the extra cellular matrix leads to the recruitment and activation of Rac1, promoting the extension of membrane protrusion at the cell front. Downstream Rac1 effectors, such as WAVE complex, induce the polymerization of actin filaments in the vicinity of the leading-edge plasma membrane, creating the pushing forces required for membrane protrusion [72].

Rac1 activity appears to be critical for tissue repair after resolution of colitis. In this connection, ELMO1 -a component with Dock1 of a bipartite GEF for Rac1- is upregulated in inflamed intestinal tissue. In dextran sulphate sodium (DSS) experimental mouse model of colitis, systemic delivery of ELMO1- lentiviral vectors attenuated colonic inflammation and promoted recovery from colonic injury via Rac1 activation [73]. Lysophosphatidic acid (LPA) also promotes mucosal repair in DSS experimental mouse model of colitis, through the activation of Gαq/PLCβ1 -induced cell proliferation, and PLCβ2/Rac1 -induced cell migration [74]. The Rac1-PAK1-Akt signaling pathway alleviates DSS-induced colonic crypt damage and acute colitis via the preservation of survival and pluripotency of Lgr5+ colonic stem cells [75]. This protective effect is inhibited by the non-muscle-myosin-II heavy chain e9 that accumulates at epithelial injury sites [75]. The cellular inhibitor of apoptosis protein 2 (cIAP2/BIRC3) also upregulates and activates Rac1, and promotes cell migration and wound healing of human colon cancer Caco-2 cells in vitro [76]. In the same vein, the expression of cIAP2 increases in regenerating intestinal epithelial cells at the wound edge of mucosal biopsies in normal human colon in vivo, and returned to normal after reepithelialization.

On the other hand, the inflammatory response consecutive to intestinal barrier alteration might also contribute to wound repair through Rac1 mobilization. Accordingly, TNFα promotes EGFR/ Src pathways activation in intestinal epithelial cells, leading to the phosphorylation of Fak, further reinforced by the Rac1-induced ROS production and phosphatases inactivation [77].

Dietary factors might also modulate Rac1 activity, and consequently might strengthen intestinal barrier activity under physiological conditions or alleviate inflammatory severity and mucosal restitution following resolution of inflammation. In this context, Rutin-rich asparagus and black beans attenuate DSS-induced colitis by modulating the colonic microbiota, resulting in improved barrier integrity and upregulation of effectors of injury repair, including Rac1 [78,79].

Chronic intestinal inflammation, e.g., ulcerative colitis, is associated with an increased risk of colorectal cancer, due to excessive reactive oxygen and nitrogen species production, pro-inflammatory cytokines release, wound repair processes, and alteration in microbiota [80]. Thus, ROS production by the NOX1 complex in intestinal epithelial cells is tightly controlled at several levels including Rac1 ubiquination/degradation (see below) or impaired functional NOX1 complex formation through Rac1 interaction with apurinic/apyrimidinic endodeoxyribonuclease 1 (APE1) [81].

## 3. Rac1 in Colorectal Carcinogenesis

### 3.1. Rac1 Overexpression and Activation in CRC

Recurrent Rac1 P29S mutation in switch I domain, leads to gain-of-function, and is identified in 4.8% of skin tumors, and 9.2% of sun-exposed melanomas [82]. This markedly contrasts with the rare identification of Rac1 activating mutations in human colorectal cancers (0.89%). With regards to Rac1 copy number, an increase is identified in 2.1% of CRC (COSMIC v90). Nevertheless, Rac1 protein proved to be overexpressed in CRC and significantly associated with tumor stage [83]. Furthermore, Rac1 protein levels were higher in colon cancer liver metastases compared with primary colon tumor, and patients with high Rac1-expressing CRC showed shorter overall survival [83,84,85]. A recent meta-analysis of 14 studies including 1793 patients demonstrates that positive Rac1 expression correlated with tumor stage, blood vessel invasion, and lymph metastasis, but not with histological differentiation. This meta-analysis indicates that Rac1 could be used as a potential marker to predict CRC prognosis [86].

The increased Rac1 accumulation in CRC might account for an enhanced accumulation of Rac1 transcripts and/or transcription, or posttranslational modifications leading to Rac1 stabilization or decreased degradation. In this concern, Rac1 transcripts are direct target of miR-320a (a tumor suppressor miRNA), whose expression is inversely associated with CRC and cell line aggressiveness. In the human colon SW620 cancer cells, miR-320a efficiently inhibits cell migration/invasion, through Rac1 downregulation [87]. Interestingly, this miRNA might also indirectly target Rac1, through downregulation of PKCγ. PKCγ -induced Rac1 phosphorylation at Ser71 promotes the invasion of A431 cells [24]. MicroRNA-142-3p and miR-106b lead indirectly to upregulation of Rac1 in colonic cell lines, promoting EMT and cell invasiveness [83,88].

Besides accumulation, overactivation of Rac1 has been demonstrated in CRC [58,89]. High levels of Rac1-GTP were evidenced in colorectal cancerous tissues compared to control mucosa and these levels were significantly correlated with TNM stages, lymph node spread, and distant metastasis as well as patient survival [89].

The compartmentalization of Rac1 constitutes another important way in the regulation Rac1 signaling and biological processes [90]. Rac1 can be recruited in different subcellular plasma membrane subdomains, i.e., junctional complexes, leading-edge promoting antagonizing activity (see above). Rac1 is also recruited to the nucleus in cell cycle-dependent manner where it also regulates transcription factor activity, and in mitochondria where it interacts with Bcl2 and triggers ROS production. Thus, one might consider a role of Rac1 mislocalization in colorectal carcinogenesis. With this in mind, neutrophil gelatinase-associated lipocalin (NGAL)/lipocalin2 is overexpressed in CRC. In human colonic cell lines, lipocalin2 overexpression triggers relocalization of Rac1 from adherens junction to cell leading edge, and thus decreases E-cadherin-mediated cell–cell adhesion and increases cell motility and invasiveness [91].

### 3.2. Rac1 Post Translational Modifications (PTMs) in CRC

#### 3.2.1. Rac1 SUMOylation 

SUMOylation is an important PTM in the regulation of Rac1 signaling (Table 1). In response to hepatocyte growth factor treatment (HGF), the small ubiquitin-like modifier (SUMO) E3-ligase, PIAS3 interacts with Rac1 and triggers SUMO conjugation at Lys 188, 183, and 184 or 186 within the C-terminal polybasic region (PBR) of Rac1. This SUMOylation is required for increased Rac1 activation and for stimulating lamellipodia, cell migration and invasion [30]. In human colorectal cancers, Rac1 SUMOylation and activation correlates with mutant TP53 status. Accordingly, mutant TP53 competes with the SUMO-specific protease 1 (SENP1) for Rac1 binding, restraining the GTPase in a SUMOylated activated form [32]. In the human non-small cell lung carcinoma H1299 cell line, this competition promotes the metastatic phenotype. Thus, the frequent *TP53* mutation identified in 43.5% of tumors (COSMIC v90) highlights the potential role Rac1 SUMOylation unbalance in CRC.

#### 3.2.2. Rac1 Ubiquitination 

Rac1 protein level is down-regulated by ligation of the protein modifiers ubiquitin and subsequent degradation by the proteasome (Table 1). Several E3 ligase, including HACE1, XIAP, c-IAP1 ubiquitinates Rac1 on Lysine residue 147, whereas FBXL19 ubiquitinates Lysine 166 [33,40,41]. It has been reported that ubiquinated Rac1 is also actively degraded in the nucleus by the proteasome [92].

In contrast to IAPs, which bind to Rac1 irrespective of its activation status, HACE1 preferentially interacts with the GTP-bound form. Notably, HACE1 targets Rac1 for degradation when Rac1 is localized to the NOX1 NADPH oxidase holoenzyme. This event blocks the ROS generation by NOX1 complex, and thereby confers cellular protection from ROS -induced DNA damage and cyclin D1-driven hyper-proliferation [34]. HACE1 activity is regulated by a negative feed-back loop involving the Ser/Thr kinase PAK1, a downstream effector of Rac1. HACE1 phosphorylation by PAK1 decreases its ability to interact with the plasma membrane and to ubiquitinate activated Rac1 [35]. HACE1 depletion is accompanied by increased total Rac1 level and Rac1 accumulation in membrane ruffles. Moreover, this depletion enhances cell migration independently of growth factor stimulation, which may have significance for malignant conversion [33]. Genetic inactivation of HACE1 in mice results in the development of spontaneous, late-onset cancer [38]. HACE1 is considered as a tumor suppressor and is frequently downregulated through hypermethylation of a CpG island in various human malignancies including colon, breast, liver, thyroid, kidney primary tumor and cell lines. In CRC, an aberrant methylation of *HACE1* promoter is observed in 28% of tumors [39], whereas *HACE1* mutation occurs in 4.4% of tumors (COSMIC v90).

#### 3.2.3. Rac1 Phosphorylation

Carcinogenesis is associated with the activation of a series of Ser/Thr and Tyr protein kinases that might affect Rac1 subcellular localization and/or activity (Table 1). Phosphorylation of serine 71 by Akt inhibits GTP-binding activity without any significant change in GTPase activity [16]. This phosphorylation enhances Rac1 interaction and cytoplasmic sequestration by the 14-3-3 proteins [93]. Phosphorylation of Rac1 at serine 71 impairs its interaction with PAK1 and Sra-1, a component of the WAVE complex involved in actin cytoskeleton remodeling, JNK and SAPK activation, but did not affect NF-kappaB pathway [16,94]. This phosphorylation proved also to be essential for FBXL19-mediated Rac1 ubiquitination and depletion [40]. At functional level, phosphorylation of Ser71 by PKCγ or PKCζ stimulates cell migration and invasiveness in vitro [24,26]. Thus, this phosphorylation represents a reversible mechanism to shift specificity of Rac1/effector coupling, and to preferentially address selected downstream pathways.

Rac1 is also phosphorylated by Erk at threonine 108 in response to EGFR activation [19]. This phosphorylation decreases Rac1 activity, partially by inhibiting its interaction with phospholipase Cγ1, which might act as a GEF-, favors Rac1 nuclear translocation and impairs Rac1-induced cell motility.

Rac1 tyrosine residue 64 is a substrate for both Src and Fak that exerts a downward regulatory effect in Rac1 focal adhesions targeting and in cell spreading [22]. The phosphomimetic Rac1 mutant Y64D displays a lower affinity for PAK1, whereas the non-phosphorylable counterpart Rac1 mutant Y64F exhibits increased GTP-binding, increased association with βPIX, and reduced binding with RhoGDI as compared with wild type Rac1.

### 3.3. RAC1b Splice Variant

The incidence of KRAS activation in CRC led us to evaluate with Dr P. Jordan (Lisbon, Portugal) the status of other small GTPases and to identify a novel splice variant of Rac1 overexpressed in human colonic tumors that we designed Rac1b [13]. The ectopic expression of Rac1b has been later reported in various human malignancies, including breast, thyroid, ovarian, pancreatic, and lung cancers [29,95,96,97,98,99,100]. Rac1b is also expressed in human inflammatory colonic mucosa [101]. The ectopic expression of Rac1b in intestinal epithelial cells of transgenic mice increases cell proliferation and migration and contributes to intestinal wound-healing after acute inflammation [14].

Rac1b variant results from inclusion of exon 3b, which leads to a 19-amino acid in-frame insertion immediately C-terminal to the switch II domain (Figure 1B). The balance in Rac1/ Rac1b levels is regulated by splicing factors, that either induce skipping of the alternative exon 3b or favor its inclusion [102,103,104,105,106]. EGFR enhances Rac1b expression in two ways; (1) EGFR triggers a cascade of phosphorylation involving PI3K/Akt/SRPK leading the activation of SRSF1 splice factor that favors exon 3b maintenance; (2) EGFR induces the ubiquitination/inactivation of hnRNP A1, a splicing regulator that promotes exclusion of exon3b [103,104,106]. The Wnt signaling pathway favors exon 3b excision via the induction of SRSF3 expression [102].

Comparative to Rac1, Rac1b exhibits a number of distinctive features. This variant is preferentially in a GTP-bound active form, due to its reduced intrinsic GTPase activity and its impaired binding to Rho-GDI [107]. Unlike Rac1, Rac1b does not activate PAK1, Akt1, JNK, nor the transactivation activity of RelB-NF-kB2/p100 [15,108,109]. The inability of Rac1b to disrupt cell-cell contacts in keratinocytes seems to be related to Rac1b failure to stimulate PAK1 [110]. On the other hand, the extra 19-amino acid sequence enhances Rac1b binding to SmgGDS, RACK1, and p120 catenin, proteins involved in cell-cell adhesion, motility, and transcriptional regulation [111]. In this connection, the Rac1b-dependent motility and spreading of the mouse mammary epithelial SCp2 cells proved to require p120^ctn^. Rac1b has also been reported to interact with Dishevelled-3 and to form a tetramer with β-catenin/TCF that is recruited to the promoter of canonical Wnt target genes [112]. In this connection, the expression of the Rac1b splice variant in intestinal epithelial cells from transgenic mice is associated with an increase in the number of β-catenin nuclear positive cells at the bottom of the crypts [14]. Rac1b also exacerbates the cellular production of ROS leading to increased expression of the Snail transcription factor, and induction of EMT and invasiveness [27,113,114]. ROS act as signaling molecules through protein oxidation, e.g., phosphatase inactivation, but also causes oxidative damage and induces genomic instability stimulating carcinogenesis. It has been recently proposed that Rac1b might be involved in the Hutchinson–Gilford progeria syndrome, a genetic disease wherein an aging phenotype manifests in childhood. Rac1b phosphorylation at Ser71 by ROCK1 facilitates its interaction with cytochrome c, and increases mitochondrial ROS production, leading to cellular senescence [27].

Interestingly, Rac1b antagonizes TGF-β-induced EMT, suggesting that Rac1b promotes a selective response to EMT inducers [15,115]. This differential effect might be related to a mutual negative regulation of Rac1/Rac1b [116].

Experimental studies revealed that Rac1b increases G1/S progression and survival of NIH3T3 cells, and is sufficient to induce the transformation of these mouse fibroblasts [117,118]. Rac1b might also be involved in later stages of carcinogenesis. Although Rac1b overexpression alone is not sufficient to drive intestinal neoplasia in transgenic mice, it enhances Apc-dependent intestinal tumorigenesis, and promotes cecum and proximal colon carcinogenesis upon chronic inflammation [14]. Thus, it is likely that Rac1b overexpression provides a proliferative advantage to cancer cells. In this concern it has been proposed that Rac1b might alleviate the oncogenic senescence induced by BRAF mutation allowing neoplastic progression [119].

In human colorectal cancers, Rac1b overexpression is associated with *BRAF* mutation, which in advanced stage is characterized by a bad prognosis [120]. Likewise, Rac1b overexpression is associated with a poor outcome of patients with wild-type *KRAS/BRAF* metastatic colorectal cancer treated with FOLFOX/XELOX chemotherapy [121]. Rac1b proves also to facilitate chemo-resistance of colon cancer cell lines to oxaliplatin through activation of NFκB signaling [122].

Selectively targeting Rac1b and/or its interaction with molecular partners, e.g., by the use of nanobodies directed against the extra 19-amino acid sequence or by the use of the corresponding cell-permeable competitor peptides, would constitute a powerful therapeutic approach for the treatment of human malignancies with Rac1b ectopic expression.

### 3.4. Rac1 Regulators & Effectors

A second level of dysregulation of Rac1 signaling pathway in CRC might involve Rac1 upstream regulators or downstream effector systems (Table 2 and Table 3).

#### 3.4.1. Guanine nucleotide Exchange Factors (GEFs)

Two classes of exchange factors were described for Rho GTPases: the classical Dbl-related exchange factors and more recently the atypical Dock family exchange factors (Table 2).

Many of these Rac1-GEFs encompass a PH domain and are activated and/or recruited to the plasma membrane leading to Rac1 activation in response to the activation of the PI3K pathway. Activation of this pathway is frequently observed in CRC, and might account for overexpression or dysregulation of receptor tyrosine kinases (e.g., EGFR, MET), activation of the nonreceptor tyrosine kinase Src, activating mutations in RAS or in PIK3CA, or downregulation of the tumor suppressor PTEN [4,17,273].

Among the Dbl-related exchange factors, as stated above, Tiam1 is generally associated under physiological conditions with Rac1-induced E-cadherin junctional complex stabilization and inversely linked with invasive potential [188]. Nevertheless, Tiam1 might be involved at different steps of colorectal carcinogenesis.

Tiam1 is a target gene of the Wnt signaling pathway [53], and the accumulation of Tiam1 transcripts is associated with the invasiveness of colorectal carcinoma cell lines in vitro [191]. Conversely, Tiam1 knockdown inhibits cell growth and invasive properties of the human colon cancer SW480 cell line in vitro, and reduces the growth of subcutaneous xenograft and the development of metastasis after intracaecal orthotopic xenograft in nude mice [192].

Tiam1 transcripts are the target of a series of microRNAs, including miR-29b and miR-21-5p. The accumulation of miR-29b is decreased in human colorectal cancers and cell lines. The ectopic expression of miR-29b and Tiam1 downregulation in human colonic cell lines inhibits epithelial–mesenchymal transition and suppresses tumor growth and metastasis in athymic nude mice [193]. Circular RNAs (CircRNAs) contain miRNA binding sites and can function as miRNA sponges. The circRNA-ACAP2 targets miR-21-5p which exhausts Tiam1. In human CRC and in the colon cancer SW480 cell line, CircRNA-ACAP2 and Tiam1 are expressed at a high level. The depletion of circRNA-ACAP2 or Tiam1, or overexpression of miR-21, inhibits SW480 cell migration and invasiveness [194].

Tiam1 transgenic mice display more invasive tumors with metastatic potential than wild-type mice, after chemical induction by 1,2-dimethylhydrazine [195]. These tumors are characterized by a marked EMT phenotype (nuclear β-catenin, decreased E-cadherin, vimentin enrichment).

Tiam1 is also overexpressed in human CRC [53,58,196], and this significantly correlated with poor prognosis and the absence of response to chemotherapy [197]. This overexpression is associated with EGFR level [198]. Accordingly, Tiam1 phosphorylation by the EGFR/Akt pathway triggers its interaction and stabilization by the 14-3-3 proteins, leading to an increased accumulation of Rac1-GTP and expression of the Wnt responsive genes cyclin-D1 and c-Myc involved in cell proliferation, [198]. Mechanistically, Tiam1 and Rac1 are components of transcriptionally active β-catenin/TCF complexes at Wnt-responsive promoters, and serve to enhance Wnt target gene transcription [213]. The interplay of Rac1/Tiam1 and the Wnt pathway is illustrated by the decrease in the number and the size of intestinal tumors following knockout of Tiam1 in APC^Min/+^ mice [53]. It is worth noting that, although Tiam1 effects on cellular migration are mediated by Rac1, Tiam1-induced resistance of colon cancer cells to anoikis is independent of the GTPase [196].

On the other hand, Tiam1 proved also to be a critical antagonist of CRC progression through inhibiting TAZ/YAP. These effectors of Wnt and Hippo signaling pathways are co-activators of the transcriptional enhanced associate domain (TEAD) proteins that regulate cell proliferation and stem cell functions. Tiam1 antagonizes TAZ/YAP (1) in the cytoplasm by promoting TAZ degradation consecutively to the interaction with βTrCP E3-ligase, (2) in the nucleus by suppressing TAZ/YAP interaction with TEADs, and the transactivation of target genes implicated in epithelial-mesenchymal transition and cell migration [274].

Other Rac1 GEFs have been involved in colorectal carcinogenesis. Vav1 overexpression is associated human colorectal tumor at advanced stage and with lymph node metastasis [200]. Vav2 mutations are identified in 4.2% of CRC (COSMIC), and have been associated with high risk of recurrence for patients with stages II and III CRC [201].

Asef1 (APC-Stimulated Guanine Nucleotide Exchange Factor 1)/Rho Guanine Nucleotide Exchange Factor 4 (ARHGEF4) is a Rac1- exchange factor stimulated by APC. Asef1 GEF stimulation by APC leads to Rac1 activation, lamellipodia formation, and increased cell migration [200]. Kawasaki et al. have identified a second Asef, termed Asef2, that shows significant structural and functional similarities to Asef1. Asef2 increases the levels of the active forms of Rac1 when co-transfected with truncated mutant APC expressed in colorectal tumor cells [275].

Consistent with previous reports [276], the levels of cytoplasmic APC in colorectal tumor cells harboring an APC mutation (SW480, SW620, HT29, Caco-2 and LoVo cells) were significantly higher than those in MDCK II and colorectal tumor cells with wild-type APC (HCT116 and SW48 cells). Similarly, the levels of Asef in the cytoplasm were also increased in colorectal tumor cells bearing APC mutations. The different subcellular localizations of Asef may determine whether Asef stimulates E-cadherin-mediated cell–cell adhesion or cell migration. Interestingly, cells coinfected with full-length Asef1 and the armadillo repeat region migrate more rapidly than cells coinfected with Asef1 and full-length APC [148]. Asef2 activated by truncated mutant APC is required for aberrant migration of colorectal tumor cells [150,275] (see Table 2). It has therefore been proposed that truncated forms of APC often found in colorectal cancer are not only deleterious due to unregulated β-catenin accumulation but may also enhance cellular metastasis due to constitutive Asef activation.

Immunostaining of ARHGEF7 (beta-PIX) is markedly increased in CRC compared with control tissues. This expression correlates with colorectal adenocarcinoma metastasis, and is associated with a shorter disease-free survival and a shorter overall survival [145]. The overexpression of ARHGEF7 in human colon cancer HCT116 and LoVo cells significantly enhanced cell migration and invasion, whereas the knockdown of ARHGEF7 in colorectal adenocarcinoma cells significantly decreased cell migration and invasion. Recently whole genome studies of colorectal metastases vs. matched primary tumors extended multistage colorectal cancer progression model with new specific for metastases components ARHGEF7 and ARHGEF33 [146]. Recurrent mutations in *ARHGEF* genes and the distribution of mutations in several *ARHGEF* genes clustered toward the RhoGEF and Plekstrin homology (PH) domains. ARHGEF7 mutations associate with worse disease-free survival [146]. These mutations were mutually exclusive with *KRAS* or *NRAS* mutations, suggesting that these may play a similar role to RAS activation. This is of special importance as patients with RAS activation do not respond well to EGFR inhibitors panitumumab and cetuximab, which may mean that metastasis with EGFR amplification and carrying an *ARHGEF7* or *ARHGEF33* mutation, may not respond to EGFR targeted therapy. On the other hand, ARHGEF7 prove to be determinants for irinotecan sensitivity of colorectal cancer cell lines [147].

The Dock (dedicator of cytokinesis) family proteins constitute another evolutionary conserved exchange factor (GEFs) for the Rho GTPases Rac1 and Cdc42, encompassing 11 related proteins gathered in four groups (A-D). Because Docks lack a Dbl domain, they are often referred to as ‘‘atypical GEFs”.

The DHR1 domain of Dock GEFs favors their recruitment to the membrane in response to PI3-kinase activation by directly binding to PIP3. The ELMO (engulfment and cell motility) scaffolding molecules binds Dock A/B group of proteins (Docks 1-5) relieve Docks autoinhibition and orchestrate their recruitment to discrete cell areas to allow polarized Rac1 activation and cytoskeleton remodeling [151].

In this context, ELMO3 transcript and protein levels are upregulated in CRC, and this overexpression is associated with tumor size, tumor differentiation, lymph node metastasis, and distant metastasis. Silencing of ELMO3 in the human colon cancer HCT116 cell line inhibits proliferation, invasion, and F-actin polymerization [277].

The cortactin-driven (CTTN) invasion by CRC cells is dependent of the activation of DOCK1-Rac1 [160]. CTTN expression in CRC correlates with depth of invasion, lymph node metastasis, and Tumor-Node-Metastasis stage [161]. Mechanistically, CTTN increases accumulation of DOCK1 and Rac1, and DOCK1 silencing partially abolishes the migration and invasion capacity induced by CTTN in SW480 colon cancer cells [160]. This observation emphasizes the potential role of DOCK1 in CTTN-mediated colorectal cell migration and invasion.

A recent, whole genome sequencing of CRC identified non-silent *DOCK2* mutations at frequencies of >7%. DOCK2 is more frequently mutated in hypermutated (MSI or *POLE*/*POLD1* driver mutations) CRC (38.0%) than in non-hyper-mutated CRC (3.9%) [130]. As regards DOCK2 expression, strong immunostaining in CRC was associated with good clinical outcome (tumor size, TNM stage, metastasis), and a longer overall survival [163]. These observations might be related to the recruitment CD8+ T lymphocytes in tumors expressing DOCK2.

Several recent studies have reported that the gut microbiome influences the efficacy of anti-PD-1/L1 immunotherapy. Accordingly, DOCK2 may participate in the immune response initiated by gut microbes, and its deficiency is most likely involved in an immune evasion mechanism of high-risk hypermutated CRC.

Other mediator of the canonical Wnt/β-catenin signaling pathway, implicated in Rac1 activity regulation, and important player in colorectal tumorigenesis and progression, is HEF1 (Human Enhancer of Filamentation 1), also known as NEDD9 or Cas-L (Cas-L adaptor molecule) [179]. NEDD9 is a member of the p130Cas family, a multi-domain skeleton protein that serves an important role in the cell signaling process via modulating invasion, metastasis, proliferation, and apoptosis, as well as mediating the hypoxia-induced migration of colorectal carcinoma cells [177,278]. NEDD9 complexes with DOCK3 to regulate Rac1 activity [9,165].

DOCK3, also referred to as modifier of cell adhesion (MOCA), was also shown to be an inhibitor of Wnt/ β-catenin signaling [279]. Moreover, multiple studies reported DOCK3 to be implicated in cancer cell invasion and migration [280]. Genome-wide association studies (GWAS) of advanced colorectal adenomas and control tissues identify a significant association of the intronic variant rs17659990 of DOCK3 with colorectal cancer risk [166].

In addition, NEDD9 is positively correlated with the expression of mesenchymal-type marker proteins such as vimentin and Zeb, while E-cadherin is opposite [178,281]. The elevated expression in colorectal cancer significantly correlated with high TNM stage and associated with poor prognosis of CRC patients [179]. Downregulation of NEDD9 by apigenin suppresses the migration, invasion, and metastasis of colorectal cancer cells [178].

The Rac1-GEF FARP2 contributes to the collective invasion of colorectal cancer cells as glandular structure maintaining apico-basolateral cell polarity. Decreased ROCK2 activity in a restricted number of cells within a cohort leads to leader cell formation. This process involves the recruitment of FARP2 to the apical junctional complex and F-actin polymerization through polarized Rac1 activation, and F-actin contractility through the Myosin II inhibition [173]. This contrasts with the ROCK pro-invasive function in other cancers, illustrating that the molecular mechanism of metastatic spread likely depends on tumor types and invasion mode.

In contrast, although the Rac1-GEFs P-REX1/2 stimulate cell migration, to our knowledge, no study has reported the implication of these GEFs in CRC.

#### 3.4.2. GTPase-Activating Proteins (GAP)/Rho GDP Dissociation Inhibitor (RhoGDI)

Conversely, Rac1 activation in CRC might result from the dysregulation of RhoGAPs (Table 2). In this connection, ARHGAP15 mRNA and protein levels are obviously lower in CRC as compared to control mucosa, and are significantly correlated with clinical stage, tumor size metastasis, vital status, and overall survival of CRC patients. The downregulation of this Rac1-specific GAP may account for the inactivation of the FOXO1 transcription factor by Akt. Overexpression of ARHGAP15 inhibits the growth, migration, and invasive properties of the human colonic HT-29 cell line, whereas opposite effects are observed in ARHGAP15-silenced LoVo cells [124].

RacGAP1 is overexpressed in CRC at both mRNA and protein levels [135]. RacGAP1 is present in the nucleus, but a diffuse distribution in the cytoplasm also occurs. Colorectal cancer patients had opposite prognoses depending on the subcellular accumulation of RacGAP1 expression. Patients with high nuclear RacGAP1 expression had poor outcomes, whereas those with high cytoplasmic RacGAP1 expression had favorable prognosis [136].

RCC2 (Regulator of Chromosome Condensation 2) is a guanine exchange factor for the RalA small GTPase and is implicated in kinetochore-microtubule function mitosis. RCC2 has been identified as a biomarker for colorectal cancer. RCC2 is a transcriptional target of TP53 -frequently mutated in MSS CRC- that proved to physically interacts and deactivates Rac1 and inhibits migration of colon cancer cells [185]. Decreased expression of RCC2 as a result of mutation in the 5’ UTR of RCC2 gene is associated with improved outcome in CRC with microsatellite instability (MSI). In contrast, weak protein RCC2 accumulation in microsatellite stable (MSS) CRC is associated with a poor prognosis [186].

Diacylglycerol kinase ζ (DGKζ) act as a critical regulator of both Rac1 and RhoA activity. DGKζ-derived phosphatidic acid activates PAK1, which phosphorylates RhoGDI, allowing for the release and subsequent Rac1 activation [282]. DGKζ protein levels were elevated approximately 3-fold in the colonic SW620 carcinoma cells compared to SW480 cells. The SW480 and SW620 cell lines are derived from primary and secondary tumors resected from a single patient. Knockdown of DGKζ expression in SW620 cells significantly reduced Rac1 activity and attenuated the invasiveness of SW620 cells in vitro [283].

#### 3.4.3. Rac1 Effectors and Signaling Pathways

Activated Rac1 interacts with a wide range of effectors that mediate signaling pathways converging to various biological functions including cytoskeleton remodeling, junctional complexes activity, cell spreading, cell motility, cell proliferation and differentiation (Figure 2, Table 3).

IQGAP1 (IQ motif-containing GTPase-activating protein 1) is a scaffolding molecule that interacts with a multitude of molecular partners [284] among them, as stated above, some components of E-cadherin junctional complexes and the guanine exchange factor Tiam1. IQGAP1 is an effector of Rac1/Cdc42 GTPases in the regulation of actin cytoskeleton dynamics and cell-cell adhesion. On the other hand, IQGAP1 inhibits intrinsic Rac1 GTPase activity, increasing Rac1-GTP pools. In human colorectal cancers, IQGAP1 is overexpressed in tumor tissues as compared with control mucosa. IQGAP1 expression is preferentially expressed at the invasion front, and especially in advanced carcinomas that invaded into the subserosa, suggesting a role of IQGAP1 in CRC invasion [231]. SUMOylation might be responsible of IQGAP1 stabilization in CRC, and in the increased proliferation and migration of colon cancer cells in vitro and xenografted in nude mice, through activating ERK, MEK, and Akt signaling pathways [285].

Although the causal link has not been investigated, IQGAP2 expression in CRC correlated positively with patient survival, while on the contrary, IQGAP3 expression levels correlated inversely with survival [233]. Up-regulation of IQGAP3 was identified in the subgroup of CRC with MSS-TP53 mutant phenotypes associated with a poor CRC-specific survival [234].

As stated above, Rac1 stimulates the Wnt/β-catenin signaling pathway, which exerts a critical role in the stemness of normal and neoplastic intestinal epithelial cells. The ROS produced by the NOX1 complex at the membrane or by the mitochondria through the Rac1/Bcl2 or Rac1/cytochrome c interactions and the downstream activation of JNK and NFkB pathways and their downstream target genes are clearly involved in EMT and cell invasiveness. Genetic deletion of Rac1 in mouse intestinal epithelial cells demonstrates the requirement of the GTPase in Wnt-induced transformation and colorectal cancer tumor initiation. The pro-tumorigenic effect was independent of β-catenin nuclear translocation and was mediated by NOX1 -induced ROS production [58].

Besides the level of ROS intracellular accumulation, the source of production and their subcellular accumulation might trigger opposing cellular responses. In this connection, Cheung et al. have demonstrated that, following APC loss, ROS produced by the NOX holoenzyme trigger intestinal epithelial cell proliferation, whereas mitochondrial ROS accumulation as a result of depletion of TIGAR—a protein that control glucose metabolism—decreases cell proliferation [286]. Interestingly, TIGAR and Rac1 expression are both controlled by the Wnt -induced Myc transactivation, suggesting that Wnt pathway integrates ROS signals to promotes cell proliferation.

Activated Rac1 also directly interacts with STAT3 to promote STAT3 phosphorylation, thus triggering EMT of CRC cells [24]. Interestingly, STAT3 proved to be overexpressed in CRC and to promote the transcription of the Nck1 adaptor molecule [216]. Nck1 serves as a scaffold for the recruitment of PAK1, Cdc42 and Rac1. Nck1 levels in CRC correlates with serosal invasion and lymph node metastasis, and cause invasiveness of CRC cell lines in vitro and enhance their metastatic potential in vivo through the activation of PAK1/Erk1 pathway.

As regards the p21-activated kinases (PAKs), they act as downstream effectors of Cdc42 and Rac1 GTPases. All PAKs are characterized by an N-terminal p21-GTPase binding domain and a highly conserved C-terminal kinase domain. These Ser/Thr protein kinases are composed of 6 members gathered in two groups. Groups I PAKs (PAKs 1-3) includes several domains that are absent in Group II PAKs (PAKs 4–6), i.e., autoinhibitory domain that overlaps with the GTPase binding domain (except for PAK5), proline rich domain, binding site for the ARHGEF7/β-PIX GEF and for the Nck adapter protein [218,219]. These proteins exert a critical role in many cellular processes, including cell morphology, survival, transcription, cell cycle progression, motility, apoptosis. PAKs are activated by both GTPase-dependent and -independent mechanisms, and mediate their biological activity through phosphorylation or acting as molecular scaffolds.

PAK1 immunostaining gradually increases during colonic carcinogenesis from normal mucosa to adenomas and adenocarcinomas [287]. PAK1 is overexpressed in primary CRC, and high PAK1 immunoexpression is associated with disease recurrence. However, there was no association with most clinicopathological parameters [288]. Song et al. reported that PAK1 and PAK4 expression are associated with CRC metastasis and infiltration, and that high PAK1 expression may indicate poor prognosis [220]. PAK1 is also overexpressed in inflammatory bowel disease and colitis associated carcinomas [289].

The cross-talk of PAK and the Wnt signaling pathway is highlighted by the inhibition of endogenous PAKs that impedes the transition of adenoma to carcinoma in an Apc-driven mouse model of colorectal cancer [290]. Similarly, PAK4 phosphorylates and stabilizes β-catenin by inhibiting its degradation. Moreover, PAK4 nuclear accumulation enhances β-catenin nuclear import and increases TCF/LEF transcriptional activity [223].

PAK5 proved also to be overexpressed in CRC. Immunostaining of PAK5 barely detectable in normal mucosa, gradually increases along the CRC progression from adenoma to adenocarcinoma and liver metastases, and correlates with tumor stage and dedifferentiation [224]. Gain and loss of function experiments of PAK5 in the SW480 colon cancer cell line revealed that PAK5 reduced cell adhesion but promoted their migration [224]. The mitochondrial localization of PAK5 and the phosphorylation of Bad protein (Bcl2-associated agonist of cell death) plays an essential in cell protection from apoptosis. PAK5 inhibits camptothecin-induced apoptosis by suppressing the activity of caspase-8 and the phosphorylation of Bad in CRC cells [291].

PAK1 might also be related to resistance to chemotherapies. Accordingly, PAK1 overexpression partially overcomes the 5-fluoro-uracile (5-FU) -induced growth inhibition of human colon cancer cells xenografted in scid mice, whereas PAK1 inhibition acts in synergy with 5-FU treatment [292]. Such PAK1-induced resistance to chemotherapies might be related to the involvement of PAK1 in DNA damage repair [218,293].

The activation of the Rac1 and Cdc42 signaling results in the formation of actin stress fibers, membrane ruffles, lamellipodia, and filopodia respectively and in cortical actin assembly. Pathways through which Rho GTPases elicit these effects are through direct interaction with members of the Wiskott–Alrich syndrome protein (WASP) family which stimulates structures such as lamellipodia and filopodia.

Rac1 -induced lamellipodia formation involves the WAVE regulatory complex and Arp2/3 complex that binds to the sides of existing actin filaments and initiates growth of a new filament. In CRC, Arp2 and WAVE2 proteins are frequently colocalized in the same cancer cells, whereas they are not detected in the normal colonic epithelial cells. Arp2 and WAVE2 were scattered throughout the cancer tissue and frequently appeared at the invasive front, where budding processes are formed. The colocalization of WAVE2 and Arp2 prove to be an independent risk factor for liver metastasis of colorectal carcinoma [291]. High mRNA levels of Arp2, is significantly associated with liver metastasis. In lines with these results, the accumulation of Wave2 and Arp2 was lower in human colonic SW480 cell line as compared to the corresponding SW620 cell line derived from the metastatic lymph node of the same primary CRC [294].

Epidemiological evidence suggests type 2 diabetes mellitus as a risk factor for cancer. In this connection, high glucose levels promote the proliferation of breast cancer cell lines through Rac1-mediated EGFR activation [295]. The search for common susceptibility genes for diabetes mellitus target organ damage and CRC pointed out several pathways, including inflammation and Wnt/β-catenin pathways [296]. Interestingly, Myc—a Wnt target gene—increases glucose transporters and glycolytic enzymes expression and mediates the Warburg effect, but also stimulates mitochondrial biogenesis [297]. Tigar transactivation by Myc might also alleviate the deleterious mitochondrial oxidative stress and ROS overproduction, consecutive to hyperglycemia-induced Rac1 activation [209,286]. Rac1 and Bcl-2 crosstalk in mitochondria to activate STAT3, promoting a permissive redox milieu for cell survival [298]. STAT3 dependent gene transcription and its nontranscriptional mitochondrial function converge to promote cell survival and likely contribute in the neoplastic progression of colorectal epithelial cells [299,300].

## 4. Conclusions, Perspectives

**Figure 4:**
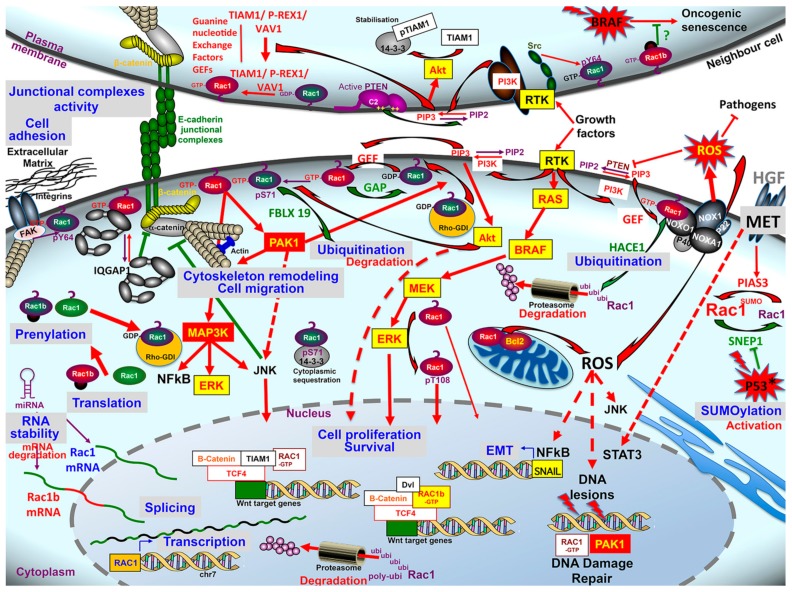
Schematic overview of Rac1 signaling dysregulation in colorectal cancer, at the levels of Rac1 itself (transcripts stability, splice variant, post-translational modifications), of its upstream regulators and interacting partners, of its downstream effectors systems, and the biological impact of these dysregulations. For details see the text.

This review illustrates the pleiotropic activity of Rac1 under physiopathological conditions and during colorectal carcinogenesis. The Rac1 signaling pathway exerts a critical role in intestinal homeostasis from crypt formation during ontogeny, to cell differentiation, polarization, and cell–cell cohesion, to maintain intestinal barrier integrity on the one hand, cell proliferation and motility to allow mucosa repair following injuries on the other. These processes are obviously reactivated and hijacked during neoplastic progression. Although Rac1 mutation proves to be a rare event in CRC, this Review unveils the different levels of dysregulation of this signaling pathway during colorectal carcinogenesis (Figure 4). The increased accumulation and activity of Rac1, its altered subcellular distribution, and the ectopic expression of the Rac1b splice variant are clearly related with increased proliferative and invasive properties of colon cancer cell in vitro, and associated to invasiveness, metastatic spread, and refractoriness to chemotherapies of human CRC. The critical role of Rac1 in colorectal carcinogenesis is further highlighted by the unbalance of Rac1 regulators (GAP, GEF, RHOGDI) and upstream and downstream effectors systems. Targeting the many pathways controlled by Rac1 has so far been a holy grail in cancer drug discovery. Promising strategies are actually devoted to target Rac1 itself, and several leads are under investigation, including nucleotide binding inhibitors, prenylation inhibitors, SUMOylation inhibitors, GEF interaction inhibitors, RhoGDI and GAP modulators, as well as PAK1 inhibitors [301,302]. In conjunction with these strategies, the ongoing development of organoid technology opens up new perspectives in precision medicine to assess individual tumor responses to these targeted therapeutic agents.

## Figures and Tables

**Table 1:** Post translational modifications (PTMs) and functional consequences.

Molecular Partners	Full Name	Function	Effect on Rac1 and Biological Impact	References Linked with CRC *
RAC1	Ras-related C3 botulinum toxin substrate 1	small GTP-binding protein, Small GTPase	Rac1 oligomerization via C-terminal domain (basic amino acid residues 183–188). Increased intrinsic GTPase activity, increased PAK1 activation [11].	[6,12]
RAC1b	Ras-related C3 botulinum toxin substrate 1, transcript variant Rac1b	small GTP-binding protein, Small GTPase	In frame insertion of an extra 19-amino acid sequence. Preferentially in a GTP-bound active form. Overexpressed in CRC. Not sufficient to initiate colorectal carcinogenesis in transgenic mice, but cooperates with Wnt signaling, and promotes colon carcinogenesis upon chronic inflammation.	[13,14,15]
**Post Translational Modifications (PTMs); Phosphorylation**				
AKT1, PKB	AKT Serine/Threonine Kinase 1	Serine/Threonine Kinase	Rac1 inhibition: Akt phosphorylates Rac1 at Ser 71 and inhibits GTP-binding activity [16].	[17,18]
MAPK3, ERK1, P44	Mitogen-Activated Protein Kinase 3	Member of the MAP kinase family, Serine/Threonine protein kinase	Phosphorylation of RAC1 at Thr108: decreased Rac1 activity, partially due to inhibiting its interaction with phospholipase C-γ1 (PLC-γ1) [19].	[18,20,21]
PTK2, FAK1	Protein Tyrosine Kinase 2	Cytoplasmic protein Tyosine kinase concentrated at focal adhesions	Phosphorylation of Rac1 at Tyr64 decreases GTP binding and Rac1 activity. Phosphorylation targets Rac1 to focal adhesions. Decreased association with βPIX, and increased binding with RHOGDI. Decreased cell spreading [22].	[23]
PRKCG	Protein Kinase C Gamma	Protein Serine/Threonine kinase activated by calcium and diacylglycerol	Phosphorylation of Rac1 at Ser71 promotes invasion of A431 cells [24].	[25]
PRKCZ	Protein Kinase C Zeta	Member of the PKC family of Serine/Threonine kinase. Independent of calcium and diacylglycerol but not of phosphatidylserine	Phosphorylation of RAC1 at Ser71 promotes proliferation, and migration of LoVo colon cancer cells via PAK1/β-catenin pathway.	[26]
ROCK1	Rho Associated Coiled-Coil Containing Protein Kinase 1	Protein Serine/Threonine kinase, activated when bound to GTP-Rho	Phosphorylation of Rac1b at Ser71 facilitates its interaction with cytochrome c. Increased mitochondrial ROS production [27].	[28]
SRC	SRC Proto-Oncogene, Non-Receptor Tyrosine Kinase	Non receptor protein Tyrosine kinase	Phosphorylation of Rac1 at Tyr64 decreases GTP binding and Rac1 activity. Phosphorylation targets Rac1 to focal adhesions. Decreased association with βPIX, and increased binding with RhoGDI. Decreased cell spreading [22].	[23,29]
**SUMOylation/deSUMOylation**				
PIAS3	Protein Inhibitor of Activated STAT 3	SUMO-E3 ligase	SUMOylation of Rac1 polybasic region (amino-acid residues 179–188) in the cytoplasm. Increased Rac1 activity and optimal cell migration in response to HGF signaling. PIAS3-mediated feedback loops controls cell proliferation and function as robust driving forces for Colitis Associated Cancer progression [30].	[31]
TP53, P53	Tumor Protein P53	Tumor suppressor. Protein containing transcriptional activation, DNA binding, and oligomerization domains	Mutant TP53 competes with SENP1 for Rac1 interaction. Protects SUMOylated Rac1 from SENP1 peptidase. Increased Rac1 activity. In CRC, Rac1 SUMOylation/activation correlates with mutant TP53 status.	[32]
SENP1	SUMO Specific Peptidase 1	Cysteine protease, deconjugates sumoylated proteins.	Mutant TP53 competes with SENP1 for Rac1 interaction. Decreases Rac1 SUMOylation and activity [32].	
**Ubiquitination**				
HACE1	HECT Domain and Ankyrin Repeat Containing E3 Ubiquitin Protein Ligase 1	E3 ubiquitin-protein ligase. Tumor suppressor. Mutated in cancers	Ubiquitinates and targets Rac1 (preferentially GTP-bound form) to proteasomal degradation. The Rac1 downstream kinase PAK phosphorylates and impairs HACE1 -induced Rac1 ubiquitination. Controls cell proliferation, cell migration, ROS production [33,34,35,36,37].	[38,39]
FBXL19	F-Box and Leucine Rich Repeat Protein 19	E3 ubiquitin ligases (member of the Skp1-Cullin-F-box family)	Ubiquitination of Lys 166 of Rac1 (preferentially phosphorylated at Ser71). Rac1 degradation by proteasome [40].	
BIRC2, c-IAP1	Baculoviral IAP Repeat Containing 2	E3 ubiquitin-protein ligase. Member of family of apoptotic suppressor proteins	Polyubiquitination of activated Rac1 at Lys147 and proteasomal degradation. Reverses mesenchymal morphology and cell migration [41,42].	[43]
XIAP	X-Linked Inhibitor of Apoptosis	E3 ubiquitin-protein ligase. Member of family of apoptotic suppressor proteins	Polyubiquitination of activated Rac1 at Lys147 and proteasomal degradation. Reverses mesenchymal morphology and cell migration [41,42].	[43]
**Bacterial Toxins-Induced PTMs**				
Toxin	Source	Rac1 PTM	Effect on Rac1 and Biological Impact	
TcdA, TcdB (toxin A, B)	*Clostridium difficile*	Mono-glycosylation of Thr35 of Rac1 (switch I).	Rac1 inactivation, actin depolymerisation, loss of cell-cell contacts and apoptosis [44]	
VopS	*Vibrio parahaemolyticus*	AMPylation of Thr35 of Rac1 (switch I).	Rac1 inactivation [45].	
YopT	*Yersinia enterocolitica*	Cysteine protease; proteolysis of the Rac1 carboxy-terminal upstream CAAX box	Rac1 inactivation. Impaired membrane interaction [46].	
VopC; DNT (dermonecrotic toxin); CNF1	*Vibrio parahaemolyticus; Bordetella dermonecrotizing; Escherichia coli*	Deamidation Gln61 of Rac1 (switch II) Gln -> Glu	Constitutive Rac1 activation (decreased Rac1 GTPase activity) [47].	
DNT (dermonecrotic toxin)	*Bordetella dermonecrotizing*	Polyamination of Gln61 (evidenced in vitro, not confirmed in vivo)	Constitutive Rac1 activation (decreased Rac1 GTPase activity). Note that DNT also deaminates Gln61 of Rac1 [48]	
ExoS (Exoenzyme S)	*Pseudomonas aeruginosa*	ADP-ribosylation of Arg 66 and Arg 68; cell line dependent (HT29 colon cancer cells sensitive; J774A.1 macrophages unsensitive)	Rac1 activation. Note that exoS ADP-ribosyltransferase activity is C-term, an antagonistic GTPase-activating domain is in the N-term of ExoS [49].	

* Highlighted references correspond to the involvement of the corresponding proteins (positively Red, negatively Green) to colorectal carcinogenesis, CRC aggressiveness, response to chemotherapy, or patient overall survival. Some discrepancies between their biological impact on Rac1 and their link with CRC might be related to the mobilization of distinct signaling pathways.

**Table 2:** Upstream effectors; RhoGAsP, RhoGDIs, GEFs.

Molecular Partners	Full Name	Function	Effect on Rac1 and Biological Impact	References Linked with CRC *
**RAC1 Regulators: RhoGAPs, RhoGDIs, GEFs, RhoGAPs, RhoGDIs**				
ARHGAP1	Rho GTPase-activating protein 1 (Rho-related small GTPase protein activator) (p50-RhoGAP)	GTPase activating protein. Negative regulation of Rho GTPases	Acting as a negative regulator for Rac1-mediated signaling [123].	
ARHGAP15	Rho GTPase Activating Protein 15	GTPase activating protein. Negative regulation of Rho GTPases	Decreases RAC1 activity. ARHGAP15 inhibits growth, migration, and invasive properties of the human colonic cell lines. Downregulated in CRC.	[124]
ARHGAP22	Rho GTPase Activating Protein 22	GTPase activating protein. Negative regulation of Rac1 and Cdc42 GTPases	Rho signaling via ROCK to ARHGAP22 inactivates Rac1, suppresses mesenchymal movement and promotes amoeboid movement [9].	
ARHGAP24, FILGAP	Rho GTPase Activating Protein 24	Rac1 specific GTPase activating protein	Filamin A targets ARHGAP24 to membrane protrusion, where it antagonizes Rac1. This leads to suppression of lamellae formation and promotion of retraction. Downregulated in CRC [125].	[126]
ARHGAP32	Rho GTPase Activating Protein 32; (Brain-specific Rho GTPase-activating protein) (RICS)	GTPase-activating protein (GAP) promoting GTP hydrolysis on RhoA, Cdc42 and Rac1 small GTPases; constitutively associated with TrkA, a high-affinity receptor for NGF	Stimulates GTP hydrolysis of RhoA and Cdc42 over that of Rac1. Components in NGF-induced cytoskeletal; regulates neurite outgrowth by modulating Rho family of small GTPases; possible role in in neural/glial cell proliferation; activity regulated by Src-like tyrosine kinases [127,128,129].	[130,131]
ARHGAP43/SH3BP1	SH3 Domain Binding Protein 1	GTPase activating protein. Negative regulation of Rac1 and Cdc42 GTPases. Partner of the exocyst complex (tethers secretory vesicles to the plasma membrane)	Stimulates the GTPase activity of Rac1 during migration. Localizes together with the exocyst to the leading edge of motile cells. Rac1 inactivation is required to organize protrusions and to drive efficient directional motility. SH3BP1 overexpression promoted invasion, migration, and chemoresistance of cervical cancer cells through increasing Rac1 activity and Wave2 protein level. This effect might be related to cross-talk between Ral and Rac1 pathways [132,133,134].	
RACGAP1	Rac GTPase Activating Protein 1	Negative regulation of Rho GTPases	Decreases RAC1 activity. Overexpressed in colorectal cancers. Nuclear localization associated with poor patient outcome.	[135,136]
ARHGDIA, Rho GDI 1	Rho GDP Dissociation Inhibitor Alpha, Rho GDP-dissociation inhibitor 1	Regulates GDP/GTP exchange of Rho GTPases: inhibits dissociation of GDP.	Regulates the GDP/GTP exchange cycle and sequesters Rho GTPases in the cytosol as an inactive pool. [137,138,139].	[140,141]
**GDP/GTP Exchanges, Guanine Nucleotide Exchanges**				
ARHGEF6/COOL-2/alpha-Pix	Rac/Cdc42 guanine nucleotide exchange factor 6 (alfa-Pix) (COOL 2) (Pak-interactive exchange factor)	Rac-specific guanine nucleotide exchange factor (GEF)	Dimerization enables its Dbl and pleckstrin homology domains to work together to bind specifically to Rac-GDP. Dimer dissociation into monomeric form allows it to act as a GEF for Cdc42 and Rac1. ARHGEF6 was identified as one of key genes and pathways in the pathogenesis of CRC (microarray data analysis) [142].	[143]
ARHGEF7/ COOL-1/ beta-PIX	Rho guanine nucleotide exchange factor 7 (Beta-Pix) (COOL-1) (PAK-interacting exchange factor beta) (p85)	Rac1-specific guanine nucleotide exchange factor (GEF)	C-terminal portion of the PH domain of COOL-1 works together with the DH domain through dimerization to mediate highly specific interaction with GDP-bound Rac1 via SH3 domain of β-Pix. Recruits Rac1 to membrane ruffles and to focal adhesions. Rac1 activation mediates cell spreading. Increased expression of ARHGEF7 in CRC associated with metastasis. ARHGEF7 mutations associate with worse disease-free survival. ARHGEF 7 is one of determinants for irinotecan sensitivity/resistance in colorectal cancer cell lines [144]	[145,146,147]
ARHGEF33	Rho guanine nucleotide exchange factor 33	Rho guanine nucleotide exchange factor (GEF)	ARHGEF33 may play a similar role to KRAS and NRAS mutations.	[146]
ARHGEF4/Asef1	Rho Guanine Nucleotide Exchange Factor 4	Guanine nucleotide exchange factor for RhoA, Rac1 and cdc42. Binding of APC activates Rac1 GEF activity	Rac1 activation. Lamellipodia formation, and increased cell migration. Mutated APC and Asef are involved in the migration of colorectal tumor cells.	[148,149]
ARHGEF29/SPATA13/Asef2	Rho Guanine Nucleotide Exchange Factor 29	Guanine nucleotide exchange factor for RhoA, Rac1 and cdc42. Binding of APC activates RAC1 GEF activity	RAC1 activation. Asef2 is required for migration of colorectal tumor cells expressing truncated APC. Asef1 and Asef2 are required for adenoma formation in Apc(^Min/+^)mice. The aberrant subcellular localization of these complexes in CRC cells may contribute to the cells aberrant adhesive and migratory properties.	[148,149,150]
DOCK1, DOCK180	Dedicator of cytokinesis protein 1 (180 kDa protein downstream of CRK) (DOCK180)	Guanine nucleotide exchange factor (GEF) The complex formation between Dock180 and Elmo1 acts as a bipartite guanine nucleotide exchange factor for Rac1.	Activator of Rac1. CrkII/Dock180/Rac1 pathway involved in integrin signaling. ELMO-DOCK180 complex is positioned at the membrane to activate Rac1 signaling and actin cytoskeleton remodeling, phagocytosis and motility. Dock1 plays critical roles in receptor tyrosine kinase -stimulated cancer growth and invasion. Dock180 activity is required in cell migration and tumorigenesis promoted by PDGFR and EGFR. The cortactin-driven (CTTN) invasion by CRC cells is dependent of the activation of DOCK1-Rac1 [151,152,153,154,155,156,157,158,159].	[160,161]
DOCK2	Dedicator of cytokinesis protein 2	Guanine nucleotide exchange factor. Predominantly expressed in peripheral blood leukocytes	Regulators of Rac1 function in adherent and non-adherent cells. Dock/ELMO complex functions as an unconventional two-part exchange factor for Rac1. ELMO binding to the SH3 domain of Dock2 disrupted the SH3: Docker interaction, facilitating Rac1 access to the Docker domain, and contributes to the GEF activity of the Dock2/ELMO complex. High mutation prevalence of DOCK2 in CRC at frequencies of >7%. More frequently mutated in hypermutated CRC. High expression of DOCK2 related with good prognosis [159,162].	[130,163,164]
DOCK3/MOCA	Dedicator of Cytokinesis 3, modifier of cell adhesion protein	Rac1 GEF. Rac1-binding domain assigned to amino acids 939 to 1323. C-term region required for optimal binding	DOCK3 activates Rac1 leading to cytoskeletal reorganization. NEDD9 complexes with DOCK3 to regulate Rac1 activity. A genome-wide significant association of rs17659990 intronic variant with CRC risk [9,165].	[166]
DOCK4	Dedicator of Cytokinesis 4	Rac1 GEF. Induces GTP loading of Rac1	DOCK4 acts as a scaffold protein in the Wnt/β-catenin pathway. DOCK4 is required for Wnt-induced Rac1 activation, TCF transcription and cell migration. DOCK4 overexpression is associated with resistance of CRC cell lines to Cetuximab and Trastuzumab [159].	[167,168,169]
DOCK7	Dedicator of Cytokinesis Protein 7	Rac1 and Rac3 specific GEF.	Activator of Rac GTPases. Regulation of microtubule networks, axon formation and neuronal polarization [170,171].	
ELMO1	Engulfment and Cell Motility 1	ELMO is a critical regulator of DOCKs1-5. Dock-ELMO complex functions as a bipartite GEF for Rac1.	ELMO binding to the SH3 domain of DOCKs allows Rac1 access to the Docker domain, and contributed to the GEF activity of the DOCK/ELMO complex [158,159,172].	
FARP2	FERM, ARH/RhoGEF And Pleckstrin Domain Protein 2	Rac1 guanine nucleotide exchange factor (GEF)	RAC1 activation. Collective invasion of colorectal cancer cells.	[173]
KALRN	Kalirin RhoGEF Kinase	Rho guanine nucleotide exchange factors (GEF)	GDP/GTP exchange, Rac1 activation. Induces neurite initiation, axonal growth, and dendritic morphogenesis. Promotes smooth muscle cells migration and proliferation. Paralog of TRIO [174,175,176].	
NEDD9	Neural precursor cell-expressed, developmentally-downregulated 9, also called HEF1 and Cas-L	Adaptor molecule, member of the p130Cas family	Skeleton protein, modulates invasion, metastasis, proliferation and apoptosis of tumor cells. NEDD9 complexes with DOCK3 to regulate Rac1 activity. Blocking Rac1 signaling suppresses mesenchymal migration and enhances amoeboid movement. Downregulation of NEDD9 by apigenin suppresses migration, invasion, and metastasis of CRC cells. Elevated expression in CRC correlates with high TNM stage and associated with poor prognosis [9,177,178].	[130,163,179,180]
PREX1	Phosphatidylinositol-3,4,5-Trisphosphate Dependent Rac Exchange Factor 1	Guanine-nucleotide exchange factors for Rac family small G proteins	Stimulation of cell migration [181,182].	
PLCG1, PLC-gamma-1	Phospholipase C Gamma 1	Hydrolysis of phosphatidylinositol 4,5-bisphosphate to 1,4,5-trisphosphate (IP3) and diacylglycerol	Critical for EGF-induced Rac1 activation in vivo. PLC-g SH3 domain acts as a Rac1 guanine nucleotide exchange in vivo. Cytoskeleton remodeling and cell migration [183].	
RCC2	Regulator of Chromosome Condensation 2	Guanine exchange factor for RalA	Interacts with Switch regions. RCC2 inhibits GEF-mediated activation of Rac1, preventing formation of multiple protrusions. RCC2 interacts with and transfers Rac1-GDP to Coro1C. Depletion of Coro1C or RCC2 causes loss of cell polarity that results in shunting migration. RCC2 is a transcriptional target of TP53, it deactivates Rac1 and inhibits migration of colon cancer cells. Decreased expression in CRC associated with a good prognosis for patients with MSI-CRC, a poor prognosis for patients with MSS-CRC [184].	[185,186]
SOS1	Son of sevenless homolog 1. Ras/Rac Guanine Nucleotide Exchange Factor 1	Guanine nucleotide exchange factor (GEF) for Ras and Rac1	Sos1 triggers Rac1 GDP/GTP exchange and activates Rac1 and JNK, leading to membrane ruffling [187].	
TIAM1	T Cell Lymphoma Invasion and Metastasis	Rac1-specific guanine nucleotide exchange factor (GEF)	Rac1 activation. Stabilization of junctional complexes. Actin cytoskeleton remodeling, membrane ruffling, cell motility, invasiveness. Neurite outgrowth. Overexpressed in CRC, associated with poor prognosis and resistance to chemotherapies [188,189,190].	[53,191,192,193,194,195,196,197,198]
VAV1	Vav Guanine Nucleotide Exchange Factor 1	Guanine nucleotide exchange factors (GEFs). Expressed in hematopoietic cells	Overexpression is associated with human CRC at advanced stage and with lymph node metastasis [199].	[200]
VAV2	Vav Guanine Nucleotide Exchange Factor 2	Guanine nucleotide exchange factors (GEFs). Ubiquitous	Mutations associated with high risk of recurrence for patients with stages II and III CRC [199].	[201]

* Highlighted references correspond the involvement of the corresponding proteins (positively Red, negatively Green) to colorectal carcinogenesis, CRC aggressiveness, response to chemotherapy, or patient overall survival. Some discrepancies between their biological impact on Rac1 and their link with CRC might be related to the mobilization of distinct signaling pathways.

**Table 3:** Interacting partners and functional consequences.

Molecular Partners	Full Name	Function	Effect on Rac1 and Biological Impact	References Linked with CRC *
**RAC1 Downstream Effectors; Nitric Oxide (NO), Reactive Oxygen Species (ROS) Production**				
NOXA1	NADPH oxidase activator 1, P67phox-Like Factor	Cytosolic subunit recruited to the plasma membrane to form the active NADPH oxidase complex (NOX1) with p22phox, NOXO1, Rac1 and NADPH oxidase1 that produces the superoxide anion	Activated Rac-1 interacts with NOXA1 in the NOX1 holoenzyme at the membrane leading to reactive oxygen species production involved in cell signaling and innate immune response [202].	
NCF2, P67-Phox	Neutrophil cytosol factor 2 (NCF-2) (67 kDa neutrophil oxidase factor) (NADPH oxidase activator 2) (Neutrophil NADPH oxidase factor 2) (p67-phox)	Rac-GTP binds to the N-terminal tetratricopeptide repeat domain of p67phox. Cytosolic subunit is recruited to the membrane to form the active NADPH oxidase complex (NOX2) with p22phox, p40phox, p47phox, Rac1 (monocytes, Rac2 neutrophils) and gp91phox that produces the superoxide anion	Regulation of phagocytic NADPH oxidase activity. Activated Rac-1 interacts with p67-Phox in the NADPH complex at the membrane, causes a conformational change in the "activation domain" in p67-Phox leading to reactive oxygen species production and innate immune response [203,204,205,206,207,208].	
APEX1/APE1	Apurinic/Apyrimidinic Endodeoxyribonuclease 1	Endonuclease. Involved in DNA repair and redox regulation of transcriptional factors	Interaction with RAC1 impairs NOX1 complex formation. Decreased ROS production [81].	
BCL2	BCL2 Apoptosis Regulator	Integral outer mitochondrial membrane protein. Inhibitor of apoptotic death	Increased ROS production. Mitochondrial oxidative stress. Although anti-apoptotic molecule, a meta-analysis suggests that Bcl-2 is for a favorable prognosis for patient with CRC [209].	[210]
CYCS	Cytochrome c	Central component of the electron transport chain in inner membrane of mitochondria	Mitochondrial ROS production. Electron transfer from cytochrome c to Rac-1 modulates mitochondrial H2O2 production. Rac1b phosphorylation at Ser71 facilitates its interaction with cytochrome c. Rac1B may be involved in Hutchinson-Gilford progeria syndrome. [27,211].	
NOS2	Nitric oxide synthase-2	Inducible by LPS and certain cytokines, generates nitric oxide mediator	Increased nitrite generation and NOS2 activity through subcellular redistribution [212].	
**Transcription (co)Factors**				
CTNNB1	Catenin Beta 1, Beta-catenin	Component of the complex of proteins that constitute adherens junctions: interacts with E-cadherins. Component of the canonical Wnt signaling pathway: In the presence of Wnt ligand or consecutively to mutations of components of the complexes that trigger its phosphorylation and degradation by the proteasome (e.g., APC), β-catenin translocates in the nucleus, and acts as a coactivator for transcription factors of the TCF/LEF family, leading to transcription of Wnt target genes (e.g., Myc, Cyclin D1, MMP7)	Rac-1 interacts with β-catenin through its polybasic region. In the absence of APC in mouse intestinal epithelium, Rac1 is not required for β-catenin nuclear localization and/or for its functional activity. In LoVo colon cancer cells (mutant APC), Rac1 phosphorylation at Ser71 by PKCZ promotes nuclear β-catenin accumulation through PAK1 activation. In NIH3T3, SW480 and HCT116 cells, Rac-1 silencing or overexpression do not influence this nuclear accumulation. In contrast upon Wnt stimulation, active Rac1 induces the redistribution of Rac1/ β-catenin protein complex from the plasma membrane to the nucleus, favors the formation of β-catenin/LEF1 complex and potentiates the transactivation of Wnt responsive genes, via Jnk -induced β-catenin phosphorylation. These discrepancies might originate from the level of Rac1 accumulation and activity, and from the cellular context [26,56,58,213,214]	[3,4]
DVL3	Dishevelled Segment Polarity Protein 3	Cytoplasmic phosphoprotein involved in Wnt signaling pathway	RAC1b interacts with Dishevelled-3 to form a tetramer with β-catenin/TCF. Transcription of canonical Wnt target genes [112].	
STAT3	Signal Transducer and Activator of Transcription 3	Transcription factor activated by receptor associated kinases	Activated Rac1 stimulated STAT3 phosphorylation on both tyrosine and serine residues. Epithelial-mesenchymal transition and invasion of CRC cells. Overexpressed in CRC [215].	[89,216]
UNKL, Unkempt	Unk Like Zinc Finger	Contributes to E3 ligase activity. Nuclear localization	Activated Rac1 interacts with UNKL and promotes ubiquitination of BAF60b, a component of SWI/SNF chromatin remodeling complexes involved in regulation of transcription and chromatin remodeling [217].	
**Kinases**				
PAK1	P21 (RAC1) Activated Kinase 1	Serine/threonine-protein kinase PAK1, belongs to the subgroup I of PAKs. Interacts with the p21-binding domains PBDs of Rac1	PAK1 is activated upon binding to GTP-Rac1. Implicated in cytoskeleton dynamics, cell adhesion, migration, proliferation, apoptosis, mitosis, DNA Damage Repair, and vesicle-mediated transport processes. PAK1 phosphorylates Bad leading to uncoupling of Bad/Bcl-2 and enhanced cell survival; effectors of cytoskeletal reorganization. Links Rac1 to JNK/MAPK pathways: phosphorylates and activates MAP2K1, RAF1. Regulates transcription through association and/or phosphorylation of transcription factors, co-regulators and cell cycle-related proteins. PAK1 phosphorylation of Snail favors its nuclear accumulation and promotes transcriptional repression of E-cadherin. Phosphorylation of NF-kB triggers nuclear translocation and the transcriptional activity of the p65 subunit. Implicated in mediating signaling from Rac1 to JNK and to actin cytoskeleton. PAK1 expression is associated with CRC metastasis [123,218,219,220,221,222].	[219,220]
PAK4	p21 protein (Cdc42/Rac)-activated kinase 4 Serine/threonine-protein kinase PAK4	Serine/threonine-protein kinase PAK4, Belongs to the subgroup II of PAKs. Interacts with the p21-binding domains PBDs of Rac1	PAK4 nuclear accumulation enhances β-catenin nuclear import and increases TCF/LEF transcriptional activity. PAK4 expression is associated with CRC metastasis.	[219,220,223]
PAK5	p21 protein (Cdc42/Rac)-activated kinase 5 Serine/threonine-protein kinase PAK5	Serine/threonine-protein kinase PAK5, belongs to the subgroup II of PAKs, mitochondrial localization	Overexpressed in CRC, correlates with tumor stage and dedifferentiation.	[224]
MAP3K1, MEKK1	Mitogen-Activated Protein Kinase Kinase Kinase 1	Serine/threonine kinase. Involved in signal transduction cascades of ERK and JNK kinase and NF-kappa-B pathways. Nuclear and post-Golgi vesicle-like compartment	Interacts with active GTP-bound Rac1. Activation of the Erk and JNK kinases *via* MAP2K1 and MAP2K4 [225].	
MAP3K10, MLK2	Mitogen-Activated Protein Kinase Kinase Kinase 10	Serine/threonine kinase. Activates MAPK8/JNK and MKK4 pathway	CRIB domain interacts with GTP-bound form of Rac1. Activates MAPK8/JNK and MKK4/SEK1 [226].	
MAP3K4, MEKK4	Mitogen-Activated Protein Kinase Kinase Kinase 4	Serine threonine kinase. Activates MAPK14 (P38alpha) and JNK pathways, but not ERK	Interacts with active GTP-bound Rac1. JNK activation [225,227].	
MAP3K11, MLK3	Mitogen-Activated Protein Kinase Kinase Kinase 11	Activates MAPK8/JNK and NF-kappaB transcriptional activity	CRIB domain interacts with GTP-bound form of Rac1. JNK activation [226].	
**Scaffolding Molecules/ Rac1 Subcellular Targeting/Cytoskeletton Remodeling, Membrane Ruffling**				
IQGAP1, P195	IQ Motif Containing GTPase Activating Protein 1; Ras GTPase-activating-like protein IQGAP1	Scaffolding molecule. Regulates dynamics and assembly of actin cytoskeleton	Activated Rac1/Cdc42, IQGAP1, and CLIP-170 form a tripartite complex; activated Rac1 recruits MTs through IQGAP1. Role in cell polarization, actin crosslinking protein, accumulates at the polarized leading edge and areas of membrane ruffling. Overexpressed in tumor tissues as compared with control mucosa; localized at invasion front [228,229,230].	[231]
IQGAP2	Ras GTPase-activating-like protein IQGAP2	Scaffolding molecule. Dynamics and assembly of actin cytoskeleton	Role in generation of specific actin structures; subcellular Rac1 localization. Expression in CRC correlates positively with patient survival [232].	[233]
IQGAP3	Ras GTPase-activating-like protein IQGAP3	Scaffolding molecule. Dynamics and assembly of actin cytoskeleton	Overexpressed in MSS TP53 mutant CRC, expression levels correlated inversely with survival.	[233,234]
SH3RF1, POSH	SH3 Domain Containing Ring Finger 1, Plenty of SH3 Domains	Scaffolding molecules for components of the JNK signaling pathway. E3 ubiquitin-protein ligase activity	Links activated Rac1 and downstream JNK kinase cascade (MLKs, MLK4/7, JNK1/2). Induction apoptotic cascade [235,236].	
SH3RF3, POSH2	SH3 Domain Containing Ring Finger 3	Scaffolding molecules for components of the JNK signaling pathway. E3 ubiquitin-protein ligase activity	Scaffold for a multiprotein complex that transduces signals from GTP-loaded Rac1 to JNK activation [237].	
CCM2	CCM2 Scaffold Protein, osmosensing scaffold for MEKK3	Involved in stress-activated p38 Mitogen-activated protein kinase signaling cascade	Binds to actin, Rac1, MEKK3 and MKK3. Role in osmoregulation [238].	
KPNA2	Karyopherin alpha2, importin α-1	Nuclear transport of proteins (binds NLS)	Interacts with Rac1 nuclear localization signal (NLS, C-terminal polybasic region). Nuclear import, (independent GDP/GTP loading), requires Rac1 activation. Nuclear Rac1 coimmunoprecipitates with numerous proteins. Overexpressed in CRC [239].	[240]
YWHA, 14-3-3	Tyrosine 3-Monooxygenase/Tryptophan 5-Monooxygenase Activation Protein	Binds to phosphoserine-containing proteins	Rac1 Ser71 phosphorylation increases affinity for 14-3-3 proteins. Interaction increases EGF -induced Rac1 activation. Cytoplasmic localisation of the complexe. Overexpressed in CRC [93].	[241]
RAP1GDS1, SmgGDS	Rap1 GTPase-GDP dissociation stimulator 1 (Exchange factor smgGDS) (SMG GDS protein) (SMG P21 stimulatory GDP/GTP exchange protein)	C-terminal poly-basic region of Rac1	SmgGDS is a GEF for RhoA and RhoC, but not for Rac1. SmgGDS interacts with RAC1 C-terminal polybasic region and triggers Rac1 nuclear translocation and degradation by proteasome. Rac1b interacts more efficiently to SmgGDS than Rac1. SmgGDS splice variants control Rac1 prenylation and membrane localization: SmgGDS-558 associates with prenylated Rac1, SmgGDS-607 associates with nonprenylated GTPases and regulates its entry into the prenylation pathway [111,242,243,244].	
ARFIP2/POR1	Arfaptin-2 (ADP-ribosylation factor-interacting protein 2) (Partner of RAC1) (Protein POR1)	Downstream effector, plasma membrane	POR1 binds preferentially to GTP-bound form of Rac1 (effector binding domain, aminoacid residues 26-48). Mediates Rac1-induced signals; membrane ruffling; regulates the organization of the actin cytoskeleton [245,246,247].	
BAIAP2, IRSp53	BAI1 Associated Protein 2	Adaptor protein; links membrane bound G-proteins to cytoplasmic effector proteins	RAC1-mediated membrane ruffling. Involved in the regulation of the actin cytoskeleton by WASF family members and the Arp2/3 complex [248].	
CTNND1/P120ctn	Catenin Delta 1/P120 catenin	Member of the Armadillo protein family. Involved in cell adhesion (binds E-cadherin) and transcription	P120 catenin interacts with the extra 19-amino acid sequence of RAC1b. Directed cell movement [111].	
CAV1	Caveolin-1	Scaffolding protein, caveolar membranes	Coronin-1C and caveolin retrieve Rac1 from similar locations at the rear and sides of the cell. In absence of fibronectin, Coronin-1C-mediated Rac1-GDP extraction and recycling to the leading edge, and maintains Rac1 cellular levels. In absence of coronin-1C, caveolin-mediated endocytosis targets Rac1 for proteasomal degradation, consecutively to engagement of the fibronectin receptor syndecan-4 [249].	
CORO1C	Coronin 1C	Member of the WD repeat protein family. Actin-binding proteins that regulate actin branching by inhibition of the Arp2/3 complex and stimulation of actin depolymerization by cofilin	Coro1C Binds Rac1-GDP. Release inactive Rac1 from non-protrusive membrane. Required for Rac1 redistribution to a protrusive tip and fibronectin-dependent Rac1 activation. Increases accumulation of activated RAC1 at the leading edge of migrating cells. Directional fibroblast migration [184,249].	
CYFIP1, SRA-1	Cytoplasmic FMR1 Interacting Protein 1	Regulates cytoskeletal dynamics and protein translation. Component of the WAVE regulatory complex (WRC), through actin polymerization	RAC1 binds to CYFIP1, initiating WASF3 complex formation involved in cytoskeleton reorganization and polymerization. Promotes invasiveness of breast prostate and colon cancer cell lines [250,251].	
FLNA	Filamin A	Actin binding protein, connects cell membrane constituents to actin cytoskeleton	Mechanical force through β1-integrins triggers apoptosis through Rac1/Pak1/p38 signaling pathway. FLNa recruits ARHGAP24 to sites of force application suppressing Rac1 activation, lamallae formation and Rac1/p38-mediated apoptosis. Down-regulated in CRC.	[252,253]
FLNB	Filamin B	Actin binding protein, connects cell membrane constituents to actin cytoskeleton	JNK activation and induction of apoptosis in response to type I Interferon [254,255].	
FMNL1, FRL	Formin Like 1	Actin polymerization, morphogenesis, cytokinesis, and cell polarity	Interacts preferentially with GTP-bound Rac1. Regulation of motility and survival of macrophages [256].	
FMNL2	Formin Like 2	Actin polymerization, morphogenesis, cytokinesis, and cell polarity	Activation of Rac1 steers FMNL2 to de novo junctional actin formation at newly formed cell-cell contacts and adherens junction formation. FMNL2 enhances proliferation, motility and invasiveness of colon cancer cell lines. Overexpressed in CRC and in liver metastases [257].	[258,259]
LRRK2	Leucine Rich Repeat Kinase 2	Member leucine-rich repeat kinase family (protein with ankryin repeat region, leucine-rich repeat domain, kinase domain, DFG-like motif, RAS domain, GTPase domain, MLK-like domain, and WD40 domain). Mutation causes dominant-inherited Parkinson’s disease	Overexpression and knockdown of LKRR2 simulates Rac1 activity. Role in maintenance of neurite morphology. Role in synaptic vesicle trafficking [260,261].	
NEDD4-1	Neural Precursor Cell Expressed, Developmentally Down-Regulated 4, E3 Ubiquitin Protein Ligase	E3 Ubiquitin Protein Ligase	Rac1 stimulates Nedd4 activity and increases ubiquitylation and degradation of the adapter protein dishevelled-1 that transduces Wnt signal downstream frizzled receptor. Maturation of epithelial cell-cell contacts. Overexpressed in CRC. [262].	[263]
PARD6A, PARD6B, PARD6G	Par-6 Family Cell Polarity Regulator Alpha/Beta/Gamma	Protein with a PDZ domain and a semi-Cdc42/Rac interactive binding (CRIB) domain. Involved in asymmetrical cell division and cell polarization	Interacts with GTP-bound Rac1. PAR6, Rac1 and atypical PKC colocalize as a ternary complex in membrane ruffles (leading edge of polarized cells during movement) [264].	
PIK3R1, P85A	Phosphoinositide-3-Kinase Regulatory Subunit 1	Regulatory subunit of PI3K	P85 binds GTP bound Rac1. Rac1 triggers P85a nuclear translocation. Activation of ERK and JNK Signaling Cascades. Downregulated in CRC [265].	[4,17,266]
PLCB2	Phospholipase C Beta 2	Hydrolysis of phosphatidylinositol 4,5-bisphosphate to 1,4,5-trisphosphate (IP3) and diacylglycerol	Rac1 engages the PH domain of PLC-beta2 and optimizes its orientation for substrate membranes. Increased PLC activity [267].	
SET	SET nuclear proto-oncogene; protein phosphatase type 2A inhibitor	Inhibits acetylation of nucleosomes (especially histone H4)	SET potentiates Rac1-mediated cell migration. Phosphorylation at Ser9 dissociates SET dimers and allows SET redistribution from nucleus to cytoplasm. The SET/activated Rac1 complex is recruited to the plasma membrane, and stimulates kinase-mediated signaling. SET enhances cell migration, EMT, and induces MYC expression in CRC cells. Overexpressed is in early-stage CRC, associated with progression and aggressiveness, and a poor outcome. Lower levels of SET in MSI CRCs compared to MSS CRC [268].	[269,270]
TOP2A	DNA Topoisomerase II Alpha	Catalyzes transient breaking and rejoining of two strands of duplex DNA to relieve torsional stress that occurs during DNA transcription and replication	Rac1 is required for DNA damage induction and subsequent activation of DNA Damage Repair following treatment with topo II inhibitors. Overexpressed in CRC. Knowdown in CRC cells decreases Akt and Erk activity, and suppresses cell proliferation and invasion [269,271].	[272]

* Highlighted references correspond to the involvement of the corresponding proteins (positively Red, negatively Green) to colorectal carcinogenesis, CRC aggressiveness, response to chemotherapy, or patient overall survival. Some discrepancies between their biological impact on Rac1 and their link with CRC might be related to the mobilization of distinct signaling pathways.
